# Incidence risk of bronchopneumonia in newborn calves associated with intrauterine diselementosis

**DOI:** 10.14202/vetworld.2020.987-995

**Published:** 2020-05-29

**Authors:** Elena Kalaeva, Vladislav Kalaev, Anton Chernitskiy, Mohammad Alhamed, Vladimir Safonov

**Affiliations:** 1 Department of Biophysics and Biotechnology, Faculty of Medicine and Biology, Voronezh State University, Voronezh, Russia; 2 Department of Genetics, Cytology and Bioengineering, Faculty of Medicine and Biology, Voronezh State University, Voronezh, Russia; 3 Laboratory of Reproductive Organs, Breast and Young Farm Animal’s Diseases, All-Russian Veterinary Research Institute of Pathology, Pharmacology and Therapy, Voronezh, Russia; 4 Laboratory of Environmental Biogeochemistry, V.I. Vernadsky Institute of Geochemistry and Analytical Chemistry, Moscow, Russia

**Keywords:** erythrocytes, leukocytes, macroelements, microelements, respiratory diseases

## Abstract

**Background and Aim:**

Macro- and micro-elements are required to ensure the normal course of biochemical processes in the development of an animal’s body. Any excess, deficiency, or imbalance in chemical elements in an animal’s body can cause the development of various latent or clinically expressed pathological conditions. Diselementosis in pregnant cows may lead to impaired embryo and fetal development, as well as reduced neonatal viability. The aim of this study was to analyze the content of macroelements and microelements in the blood serum of both calving cows and their calves to evaluate the relationship between indicators of mineral metabolism in the mother and newborn and to establish what role separate chemical elements play in making newborn calves more prone to bronchopneumonia.

**Materials and Methods:**

The content of potassium (K), sodium (Na), iron (Fe), Copper (Cu), Zinc (Zn), Strontium (Sr), arsenic (As), nickel (Ni), cobalt (Co), chromium (Cr), molybdenum (Mo), and selenium (Se) in the blood serum of 33 pregnant cows between 239 and 262 days of gestation and their 33 1-day old calves was determined using the Shimadzu AA6300 (Japan) atomic adsorption spectrophotometer. Calcium (Ca) and magnesium (Mg) content was determined using ion-selective electrodes from the Olympus-400 analyzer (Beckman Coulter, USA). During the 1^st^ month of life, all calves in the sample set had some sort of respiratory diseases and seven of the calves had bronchopneumonia. Retrospectively, the samples of adult and newborn animals were divided into two groups each: Dams I – cows whose calves had uncomplicated bronchitis (n=26); Dams II – cows whose calves got bronchopneumonia (n=7); and Newborns I – calves with uncomplicated bronchitis (n=26); Newborns II – calves with bronchopneumonia (n=7).

**Results:**

The content of Ca, Mg, K, Na, Mo, and Se in dams in both groups of cows was within the reference range; the concentrations of Fe and Ni were higher than the reference range; and the concentrations of Cu, Zn, As, Co, and Cr were lower than the reference range. There were no significant differences in elemental status between the Dams I and Dams II groups. In newborn calves, the concentration of Ca and Mo corresponded to the reference range; the concentrations of Mg, Fe, Co, and Ni in both groups exceeded the reference range; and the concentrations of Cu, Zn, As, Cr, and Se were lower than the reference range. Results highlighted that there was a tendency to decrease concentration of Fe, Mo, and Se and a significant increase in the Ni concentration in calves of the Newborns II group compared with calves of the Newborns I group. It was also found that Zn, Co, Cr, and Mo actively accumulated in the body of newborn animals while the transplacental transfer of Cu, As, and Sr was limited; and transfer of Se and Ni was regulated by concentration ratios in the blood of the mother and the fetus. The excessive concentrations of Ni and Fe in the blood serum of cows and calves and the imbalance in the ratio of elements Fe–Cu–Zn, Fe–Cu–Co negatively affected erythropoiesis, formation of the immune system, and antioxidant status of the fetus and newborn. These changes were considered to be risk factors for the development of bronchopneumonia in calves.

**Conclusion:**

An excess of serum Fe and Ni and deficiency of Cu, Zn, As, Co, and Cr in cows during the gestation period can lead to similar impairments of the mineral status in newborn calves. At the systemic level, dyslementosis in combination with the influence of other adverse factors, can lead to an increased load on the respiratory and hematopoietic systems of calves during postnatal adaptation and can subsequently cause a decrease in the natural resistance of calves and development of bronchopneumonia.

## Introduction

Macro- and micro-elements are necessary to ensure the normal course of biochemical processes in the bodies of animals. Twenty-two chemical elements are considered essential for mammals; seven of them are macroelements (calcium [Ca], phosphorus, potassium [K], sodium [Na], chlorine, magnesium [Mg], and sulfur), and 15 are trace elements (iron [Fe], iodine, zinc [Zn], copper [Cu], manganese, cobalt [Co], molybdenum [Mo], selenium [Se], chromium [Cr], tin, vanadium, fluorine, silicon, nickel [Ni], and arsenic [As]) [1,2]. An excess, deficiency, or imbalance of chemical elements in the animal organism, referred to in the biological literature as “dyslementosis” [3], are usually accompanied by the development of various latent or clinically expressed pathological conditions. In farm animals, dyslementosis is usually caused by geochemical environmental conditions or impaired absorption of chemical elements from water and feed. The Voronezh region is one of the southern regions of the Central Federal District of the Russian Federation. It is located in the central zone of the European part of the country and it is a part of the Central Russian Province. Geologically, the territory of the Voronezh region is located in the southeastern part of the Voronezh crystalline massif (VCM), a large Precambrian platform-type structure. An intense manifestation of sulfide-bearing ultramafite-mafite magmatism, associated with deposits and numerous manifestations of sulfide-Cu-Ni ores, is associated with Precambrian formations in the southeast of the VCM. Deposits and manifestations of non-metallic mineral resources can form rocks of the sedimentary complex. The quality indices of drinking water in the region are determined by natural factors (an increased concentration of iron, manganese, boron, and hardening salts in the water). In most cases, the quality of groundwater in the Voronezh region is determined by either increased water hardness (up to 20 mg-eq/dm^3^) due to the presence of the carbonate component in water-bearing formation, or a significant amount of iron (from 0.3 to 6.8 mg/dm^3^ [1-26 marker and cell (MAC)]) and manganese (0.55-1.10 mg/dm^3^ [5-10 MAC], sometimes up to 25 MAC). Other heavy metals in concentrations exceeding MAC were not detected in the water [4]. Soil and plants in the Voronezh region are usually poor in iodine, Zn, and Mo [5]. Water and plants are the sources of mineral elements for cows. Excess or deficiency of minerals in water and plants affects the mineral status of animals.

It is known that dyslementosis in pregnant cows can lead to the impaired development of embryos and fetuses and a decrease in the viability of newborns [6,7]. Even subclinical disorders of mineral metabolism in pregnant cows can adversely affect the elemental status and health of offspring [8]. Unfortunately, the question of assessing the mineral status of newborn calves has not been studied enough; monitoring is often carried out using reference ranges established for adult animals, without taking into account the regional and pedigree characteristics of cattle.

Respiratory diseases in young cattle remain one of the most common problems of industrial animal husbandry [9-11]. They account for up to 75% of total morbidity and over 50% of loss in cattle cases [12]. For improvement in prevention measures and treatment of respiratory diseases in calves, it is vitally important to assess risk factors associated with impairment of the elemental status in the mother and fetus. Optimization of indices is needed for the system of reference range in the mineral metabolism of cattle to adequately interpret the results of studies and diagnoses [13]. Deficiency or excess of some mineral elements may be the cause or result of respiratory diseases. So, the search for bio element-markers in respiratory diseases to be used for routine clinical practice is important [14-16].

The aim of this study was to analyze the content of macro- and micro-elements in the blood serum of pregnant cows and their calves to identify the relationship between the indices of mineral metabolism in the mother-newborn system. Based on these findings, it can be established what role individual chemical elements play in making newborn calves more prone to the development of bronchopneumonia.

## Materials and Methods

### Ethical approval

Blood samples were collected as per standard sampling procedures without any harm to the animals. Approval from the Federal Service for Veterinary and Phytosanitary Surveillance of the Ministry of Agriculture of Russian Federation was not required; the study did not affect normal animal physiology.

### Study location and period

The studies were performed in “Voronezhpishcheproduct. Ltd” Novousmansky district of the Voronezh region, Russia during the spring 2015.

### Animal materials and study design

Pregnant, red-motley Holstein breed cows (n=33) and their calves (n=33) were used in the present study. The calves were kept in a dispensary with 5-6 heads per cage for 10-20 days. Newborn calves received colostrum from their mothers 3 times a day. For the first 10 days, colostrum (then milk) was administered in the amount of one-tenth of the animal’s total weight. To assess the calves’ health status, body temperature, cardiac rate and respiration rate, presence/absence of diarrhea, cough, nose discharge, and discharge from the eyes were all determined. Behavioral changes, sucking reflex, activity, and appetite were also taken into consideration, and thorax auscultation was carried out. During the 1^st^ month of life, inflammatory diseases of the upper respiratory tract (bronchitis, and tracheobronchitis) of varying severity were found in all of the calves. In some cases, self-recovery in the animals was observed when accompanied with proper care and feeding. Inflammation of the upper respiratory tract complicated by bronchopneumonia was also recorded in seven calves.

### Collection of samples

Blood samples were collected from cows on days 239 to 262 of gestation and from their calves 24 h after birth. This was carried out in the morning hours before feeding using jugular vein puncture. Vacuum test tubes without anticoagulants were used to obtain blood serum.

### Biochemical analysis

Concentration of microelements was determined from the blood serum of the animals using the Shimadzu AA6300 (Japan) atomic adsorption spectrophotometer, while Ca and Mg content was determined using ion-selective electrodes of the Olympus-400 analyzer (Beckman Coulter, USA).

### Statistical analysis

Statistical analysis of the research results was carried out using the Stadia 7.0 Professional (InCo, Russia) and MedCalc for Windows, version 17.5.3 (MedCalc Software, Ostend, Belgium) software packages. Retrospectively, samples of adult and newborn animals were divided into two groups each: Dams I – cows, whose calves experienced uncomplicated bronchitis development (n=26); Dams II – cows, whose calves caught bronchopneumonia (n=7); and Newborns I – calves with uncomplicated bronchitis development (n=26); Newborns II – calves with bronchopneumonia (n=7).

The data are presented in the format of arithmetic mean±standard deviation and the medians of indicators are given. A hypothesis regarding the feature distribution normality was tested using Kolmogorov’s criterion and the ω^2^ and Chi-square Pearson’s criteria. Feature medians were compared using the Wilcoxon W-test. Receiver operating characteristic (ROC) analysis according to the DeLong *et al*. method [17] was used to detect predictors of bronchopneumonia in calves. Correlations between indicators were identified using Spearman’s coefficient (r_s_). The null hypothesis was rejected for all statistical processing methods at p<0.05.

## Results and Discussion

It was found that the content of Ca, Mg, K, Na, Mo, and Se in down-calving cows in both groups was within the reference ranges established for this breed and age [18-20]. The concentrations of Fe and Ni in the blood serum of adult animals were higher than the reference values while the concentration of Cu, Zn, As, Co, and Cr was lower (Table-1). Mineral metabolism indices in cows were characterized by a wide range of variation (Table-1), and therefore, there were no statistically significant differences between the Dams I and Dams II groups for the content of macro- and micro-elements. At the trend level (0.05<p<0.1), a higher content of Cu and Ni was found in cows of the Dams II group compared to those of the Dams I group.

**Table-1:** Macroelements and microelements content in blood serum of the down-calving cows.

Indicator	Dams I		Dams II	
	
	Range of deviation (min-max)	M±s_x_, Me	Range of deviation (min-max)	M±s_x_, Me
Calcium, mmol/l	2.48-3.10	2.76±0.16 Me=2.75	2.61-2.75	2.67±0.05 Me=2.67
Magnesium, mmol/l	0.87-0.92	0.89±0.02 Me=0.90	0.84-0.93	0.90±0.03 Me=0.91
Potassium, mmol/l	3.80-5.89	4.65±0.57 Me=4.47	3.84-7.62	4.71±0.95 Me=4.44
Sodium, mmol/l	95.6-164.3	127.6±15.6 Me=127.8	122.3-142.8	132.2±7.1 Me=132.7
Iron, mg/l	1.50-4.70	2.85±0.99 Me=2.85	1.40-4.00	2.65±0.92 Me=2.50
Copper, mg/l	0.29-0.68	0.50±0.10 Me=0.48	0.39-0.85	0.59±0.16 Me=0.55^trend^
Zink, mg/l	0.11-7.74	0.58±1.51 Me=0.15	0.13-18.25	2.75±6.84 Me=0.17
Strontium, mg/l	0.35-3.27	0.95±0.67 Me=0.74	0.27-1.22	0.81±0.36 Me=0.85
Arsenic, μg/l	14.3-64.2	37.1±15.1 Me=40.1	4.5-113.6	51.0±37.3 Me=36.8
Nickel, mg/l	0.22-2.90	1.02±0.54 Me=0.93	0.53-2.23	1.31±0.62 Me=1.26^trend^
Cobalt, μg /l	10.3-38.1	22.1±6.8 Me=21.3	14.5-29.7	20.8±5.6 Me=18.9
Chromium, μg/l	0.1-3.9	0.35±0.80 Me=0.10	0.1-0.2	0.13±0.05 Me=0.10
Molybdenum, μg/l	0.4-3.2	1.38±0.71 Me=1.35	0.9-2.6	1.67±0.72 Me=1.40
Selenium, μg/l	35.2-153.1	68.0±31.2 Me=57.9	39.3-188.2	90.6±62.6 Me=59.1

^trend^=Differences from the adults I group at the level of statistical trend (0.05<p<0.01)

The increased concentration of Fe in the blood serum of cows may be due to an excess of intake in drinking water. It is known that the mechanisms regulating the metabolism of Fe stimulate its accumulation, but does not control the removal of excess iron from the body [6]. As not all of the iron entering the bodies of cows can be adequately utilized, its concentration within blood samples exceeds physiological values (Table-2).

**Table-2:** Standards of mineral elements content in the blood serum of cows and newborn calves.

Indicator	Adult animals (standard)	Newborn animals (standard)
Calcium, mmol/l	2.50-3.13	1.81-3.41
Magnesium, mmol/l	0.82-1.23	0.79-0.95
Potassium, mmol/l	4.00-6.00	4.10-5.70
Sodium, mmol/l	127.0-153.0	12.0-151.0
Iron, mg/l	0.96-2.01	1.00-1.30
Copper, mg/l	0.80-1.20	0.48-0.79
Zink, mg/l	3.00-5.00	1.00-1.50
Strontium, mg/l	No standard	No standard
Arsenic, μg/l	50.0-200.0	50.0-200.0
Nickel, mg/l	0.10-0.50	0.10-0.50
Cobalt, μg/l	30.0-50.0	28.0-38.5
Chromium, μg/l	4.00	4.00
Molybdenum, μg/l	<10.0	<10.0
Selenium, μg/l	40.0-70.0	65.0-87.0

Ni, which enters the body of animals mainly with food (drinking water makes an insignificant contribution to its accumulation), usually accumulates in tissues [21]. The increased Ni content in the blood serum of pregnant cows may have been associated with the high element content in the soils of the region and plant feeds [5]. An excess of Ni in the blood serum of animals adversely affects the balance of Cu, Zn, and Fe in the body [22,23] and depresses energy-dependent processes [24]. A negative correlation was found between the Ni content and the average hemoglobin concentration in the erythrocytes of adult animals (r_s_=−0.56, p=0.002). Moreover, the Kruskal–Wallis test highlighted a significant influence of increased Ni concentration in the blood on MCHC (k=4.01; p=0.04), which indicates the contribution of Ni to the impairment of erythropoiesis in pregnant cows.

Cu deficiency in cattle is a widespread issue in many regions of the world [25]. In the cows examined in this study, Cu deficiency was exacerbated by the excess Ni content in the body. It is known that Ni exhibits antagonist properties against Cu [23], by preventing its normal absorption and implementation in various biological functions. In turn, the Cu deficiency observed in the Dams I and Dams II groups caused hyperferremia, as Cu regulates the normal absorption of iron [26]. Cu deficiency was also associated with a decrease in the monocyte count in the peripheral blood of cows (Mon=1.0% in the Dams I and Dams II groups with a reference range of 1.5-3%). Monocytopenia negatively affects the functioning of the innate immune system which, in turn, reduces the body’s resistance to invading pathogens.

Deficiency of Co in ruminant animals leads to the impaired synthesis of Vitamin B_12_ and the maturation of erythrocytes [27,28]. Earlier, it was shown [29] that the subclinical deficiency of Cu and Co in pregnant cows leads to the development of macrocytic hyperchromic anemia and impairs normal supply of oxygen to the mother and fetus organisms. As deficiency in cows also inhibits hematopoiesis [30,31]. A correlation has been revealed between the As level and the mean cell volume (r_s_=0.36; p=0.03), which indicates the role of this trace element in the regulation of erythropoiesis in adult animals.

Lower concentrations of Zn and Cr (III) in the blood serum of pregnant cows can impair carbohydrate metabolism, delay fetal development [32], and increase susceptibility to infectious diseases and stress. These effects are associated with the biological functions of these elements in the body. For example, Zn is a component and/or activator of enzymes [33,34] which is necessary for the synthesis of steroid hormones [35]; it also affects the functional activity of the immune system [36,37]. Cr, being a cofactor of a number of enzymes, exhibits immunosuppressive properties under stress conditions [38,39] and regulates glucose metabolism [40,41]. An imbalance in the Cu–Zn–Fe system in pregnant cows, which is usually in a state of dynamic equilibrium [42], against the background of excess Ni and deficient As, is the main cause of impairments of erythropoiesis, the functioning of antioxidants and immune systems, and the deficiency of Co and Cr which enhances its negative effects.

The elemental status of a newborn calf is determined by the status of the mother cow during gestation [26] and the content of macro- and micro-elements in the colostrum. However, it has been shown [43] that not all micronutrients penetrate equally well through the fetoplacental barrier. Some of them are only able to accumulate in the mother or calf, and the regularities of metabolism common to all minerals in the mother-fetus system, therefore, do not exist. In newborn calves, only the content of Ca and Mo in the blood serum corresponded to the reference ranges (Table-3). The concentrations of Mg (insignificantly), Fe, Co, and Ni (significantly) exceeded the reference ranges in both groups and the content of Cu, Zn, As, Cr, and Se sat below the reference ranges [18-20]. In the calves of the Newborns II group, it was highlighter that there was a higher serum content of Ni (p<0.05) and decreased concentrations of Fe, Mo and Se (0.05<p<0.01) present in comparison to calves of the Newborns I group. According to the results of the ROC analysis, predictors of bronchopneumonia were not detected among the studied macro- and micro-elements in adult animals and their offspring.

**Table-3:** Macroelements and microelements content in blood serum of the newborn calves.

Indicator	Newborns I		Newborns II	
	
	Range of deviation (min–max)	M±s_x_, Me	Range of deviation (min–max)	M±s_x_, Me
Calcium, mmol/l	2.79-3.12	2.95±0.93 Me=2.95	2.70-3.13	2.96±0.15 Me=2.94
Magnesium, mmol/l	0.98-1.07	1.02±0.02 Me=1.02	0.98-1.05	1.03±0.02 Me=1.03
Iron, mg/l	0.50-31.00	6.04±6.91 Me=3.40	1.00-6.00	2.61±1.71 Me=2.10^trend^
Copper, mg/l	0.14-1.09	0.33±0.20 Me=0.29	0.11-0.57	0.36±0.18 Me=0.38
Zink, mg/l	0.7-4.22	0.66±1.11 Me=0.25	0.12-1.48	0.59±0.55 Me=0.35
Strontium, mg/l	0.06-2.14	0.38±0.45 Me=0.26	0.13-0.87	0.35±0.26 Me=0.31
Arsenic, μg/l	3.3-172.2	41.7±42.9 Me=28.4	8.7-126.4	41.8±40.1 Me=30.4
Nickel, mg/l	0.10-3.85	1.07±0.96 Me=0.96	0.05-3.17	1.74±0.39 Me=1.93*
Cobalt, μg/l	27.4-241.9	69.1±46.3 Me=61.1	24.00-102.1	63.2±30.0 Me=72.9
Chromium, μg/l	0.20-13.1	1.38±2.71 Me=0.60	0.2-2.6	0.74±0.84 Me=0.50
Molybdenum, μg/l	1.1-47.1	9.04±10.88 Me=5.40	1.0-45.2	8.83±16.12 Me=3.30^trend^
Selenium, μg/l	20.8-157.1	54.9±31.1 Me=48.1	27.6-106.7	45.6±28.3 Me=37.6^trend^

* =Differences from the newborns I group are statistically reliable (p<0.05), trend=Differences from the newborns I group at the level of the statistical trend (0.05<p<0.1)

**Figure-1:**
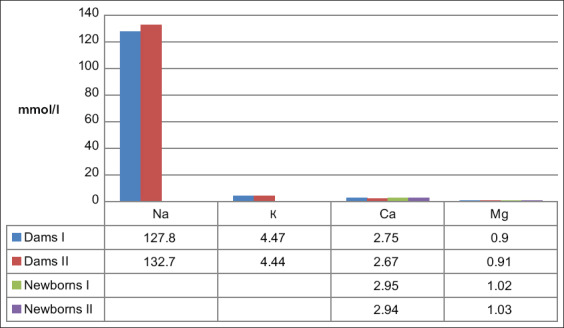
Macroelements content in the blood serum of down-calving cows and newborn calves (median indicator values).

A slight excess of Mg in the blood serum of newborn calves in comparison with the reference range established for this breed and age (Figure-1 and Table-3) did not adversely affect their condition. Mg concentrations in the calves of the Newborns I and Newborns II groups were not beyond the “adult” reference range and did not differ from the concentrations observed in their mothers, indicating that there was a free transplacental transfer of this element and an establishment of dynamic equilibrium in the mother-fetus system.

**Figure-2:**
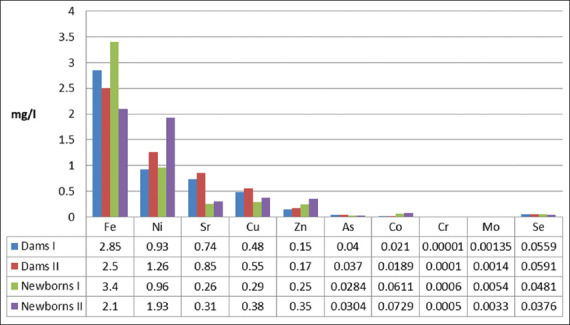
Microelements content in the blood serum of down-calving cows and newborn calves (median indicator values).

It was found that the iron content in the blood serum of the calves of the Newborns I group was higher than that of their mothers and in calves of the Newborns II group, it was lower (Table-3 and Figure-2). However, in all of the groups, the studied parameters exceeded the reference range established for this breed and age (Table-2). In general, there were no restrictions for transplacental transfer of iron, although it is possible that during gestation in animals of the Dams II and Newborns II groups, iron metabolism and/or its accumulation in the fetus could have been impaired. It is also possible that the rate of replacement of fetal hemoglobin by adult hemoglobin in the calves of the Newborns II group was lower than that of the calves in the Newborns II group, and therefore, a massive release of iron from the destroyed erythrocytes on the 1^st^ day of life was not observed. Partial excess of iron could have been included in the composition of hemoglobin, as indirectly indicated by a positive correlation between the concentration of Fe in the blood serum of calves and the mean concentration of hemoglobin in the erythrocytes (r_s_=0.32; p=0.04). There was severe microcytosis (Mean corpuscular volume=41.0 and 40.0 mm^3^) in the calves of the Newborns I and Newborns II groups, respectively; the reference range was 52.8-62.2 mm^3^ and an increase in MCHC could have been compensation for the insufficient functional viability of erythrocytes.

Zn actively accumulated in the bodies of calves under conditions of deficiency in the mother cows, and Zn level in the blood of newborns exceeded that in adult animals. This may indicate an effective transfer of this microelement through the placental barrier to meet the requirement of the fetus. However, due to the low Zn content in cows, its deficiency in calves was not compensated. It was found that newborn calves have a higher requirement for iron and Zn compared to adult animals [44]. Zn, which is considered toxic to adult animals (more than 500 mg/kg d.w.), is considered normal for young animals [45]. Thus, the existing reference ranges for the content of iron and Zn in the blood serum of newborn animals (Table-3) may require revision.

Regarding the distribution of Se and Cu between the organisms of the mother and the fetus, consensus was not reached. For example, Graham *et al*. [46], House and Bell [47], and Ceballos-Marquez *et al*. [48] believed that there is an active transfer of Se and Cu from mother to fetus, limited solely by the needs of the latter. According to several authors [49], the transmission of Se to the fetus, and then to the newborn calf, occurs even when mother cows are deficient in it, to provide the necessary levels for the newborn. Pavlata *et al*. [50] indicated that transplacental transfer of Cu is partially limited; hence, the concentration of Cu in the blood serum of calves does not exceed 60% of its content in mothers. It was found that the level of Cu in the blood serum of newborn calves was lower than that of their mothers (Figure-2 and Tables-1 and 3). This may be due to its deficiency in cows and an insufficient content of caeruloplasmin, which is the main carrier of Cu in the blood of calves [51]. In our opinion, it is believed that there was a partial restriction of the transfer of Cu from mother to fetus, as the concentration of Cu in the blood serum of adult animals was deficient but exceeded that of the calves. The correlation between the level of Cu and monocyte count in calves (r_s_=0.46, p=0.007) may indicate that there is a contribution of this element to the formation of the phagocytosis system in newborn animals. Thus, a deficiency of Cu can mediate a decrease in the resistance of newborn animal to infection through inhibition of the monocyte-macrophage system.

In newborn calves, Se deficiency was also detected, although its content in the blood serum of cows was within the reference range. In ruminant animals, the transmission of Se from mother to newborn occurs through the placenta and colostrum, while transplacental transfer is more effective [37,43,52,53]. The lack of Se in newborn animals may be due to the fact that this element is a component of antioxidant protection and is actively consumed under oxidative stress conditions during birth. The low content of Se in the calves of the Newborns II group (less than the age and adult reference range) can indicate either a more active consumption or a restriction of metabolism in the mother-fetus system. Newborn calves with hyposelenosis are known to be more susceptible to infections of the gastrointestinal and respiratory tracts [54]. The relationship between the content of Se and monocyte count in the circulating blood of calves (r_s_=0.45, p=0.007) indicates deficiency of this element in reduced resistance to infectious diseases. An excess of iron in the blood serum of calves can adversely affect Se levels, since Fe^3+^ forms low-solubility complexes with Se and reduces the absorption rate of the latter [55].

The role of the above-mentioned inorganic elements in the body and their metabolism in the mother-fetus system is fairly well understood and described in the literature, but the distribution of other trace elements between mother and newborn is less studied.

It was found that intensive transfer of Sr from mother to newborn did not occur, and the content of Sr in the blood serum of newborn calves was noticeably lower than that of their mothers (Tables-1 and 3 and Figure-2). This may have been caused by Sr accumulation and retention in bone tissue. A certain dynamic equilibrium is established between the bone and serum Sr fractions, and the active accumulation of Sr in the body occurs only with an increase in bone mass during the period of active growth of skeletal system [56].

The Ni content in all of the groups of animals exceeded the established reference ranges but was higher in calves with complicated bronchitis and in their mothers compared to the animals of the Newborns I and Dams I groups. The resulting negative correlation between the Ni concentration and lymphocyte count in newborn calves (r_s_=−0.35, p=0.03) may indicate an immunosuppressive effect of increased concentrations of this metal. At the same time, Ni concentration was positively correlated with the monocyte count in the peripheral blood of calves (r_s_=0.31, p=0.04), which indicates its stimulating effect on the phagocytic system of blood. Thus, an excess of Ni may contribute to an imbalance in the lymphocytic-monocytic system, which negatively affects resistance to infectious agents.

The concentrations of Co, Cr, and Mo in the blood serum of newborn calves exceeded those in their mothers (Tables-1 and 3). Based on the data obtained, it can be assumed that these heavy metals are actively transferred through the placenta and accumulate in the fetus, regardless of their concentration in the mothers’ bloodstream.

As deficiency in newborn calves, in contrast to adult animals, was more pronounced in the leukocyte blood count. Correlations between the As content and percent of segmented neutrophils (r_s_=0.35; p=0.03) and monocytes (r_s_=0.37; p=0.02) were found. The parameters of the leukogram in the calves were at the lower boundary of the age reference range (segmented neutrophils =32.0 and 38.0; monocytes =0.0 and 0.0 in the Newborns I and Newborns II groups, respectively), which could lead to weakening of the immune defense.

In both newborn calves and adult animals, the main systemic effects of dyslementosis included an excess of Ni, deficiency of As, and a shift of balance in the Cu–Zn–Fe system. These changes induced hematopoiesis disorders which led to the onset of microcytic hypochromic anemia [29] and functional insufficiency of the immune and antioxidant defense systems which negatively affected resistance to infectious diseases.

## Conclusion

Dyslementosis in cows during the gestation period, which is characterized by an excess of serum Fe and Ni and deficiency of Cu, Zn, As, Co, and Cr, can lead to similar impairments of the mineral status in newborn calves. The transplacental transfer of Cu, As, and Se from the mother to the fetus or its accumulation in the colostrum was partially limited, and therefore, concentrations of these elements in the blood serum of newborn calves were below reference range and lower than the corresponding levels of these elements in their mothers. Heavy metals (Ni, Co, Cr, and Mo), on the contrary, were actively transferred across the placenta. These metals were significantly accumulated in calves, regardless of the levels of these metals in the mother cows. Their content in the blood serum of day old calves exceeded that in adult animals, and the concentrations of Ni and Co were higher than the physiological level.

The wide range of variation in the study parameters, the complex system of relationships between them, and the variety of compensatory mechanisms at the organism level significantly complicated the analysis and interpretation of the role that individual trace elements play in the formation of susceptibility to a particular pathology. An analysis of the relationships in the mineral metabolism system suggested that excess Ni, deficient As, and imbalances in the Fe–Cu–Zn and Fe–Cu–Co triads in mother cows and newborn calves may be a potential threat to their health. These disorders can negatively affect erythropoiesis while also impairing the balance of the lymphocyte-phagocytic system and the prooxidant-antioxidant system. This allows them to be considered risk factors that are involved in the complications of respiratory diseases in calves.

At the systemic level, dyslementosis in combination with the influence of other adverse factors, can lead to an increased load on the respiratory and hematopoietic systems of calves during postnatal adaptation and can subsequently cause a decrease in the natural resistance calves have for the development of bronchopneumonia.

## Authors’ Contributions

EK: The idea of manuscript, literature search, and wrote the manuscript draft. EK and VK: Performed the statistical analyses. VK: Conceived and designed the study. AC and MA: Collected samples and performed the experiments. MA and VS and AC: Performed the laboratory analyses. AC and VS revised the manuscript critically. All authors read and approved the final manuscript.
